# The Integration of Spring and Winter Wheat Genetics With Agronomy for Ultra-Early Planting Into Cold Soils

**DOI:** 10.3389/fpls.2020.00089

**Published:** 2020-02-20

**Authors:** Graham R.S. Collier, Dean M. Spaner, Robert J. Graf, Brian L. Beres

**Affiliations:** ^1^Department of Agricultural, Food, and Nutritional Science, University of Alberta, Edmonton, AB, Canada; ^2^Lethbridge Research Centre, Agriculture and Agri-Food Canada, Lethbridge, AB, Canada

**Keywords:** wheat, seeding, early, grain yield, cold tolerance, stability, great plains

## Abstract

Early seeding has been suggested as a method of increasing the grain yield and grain yield stability of wheat (*Triticum aestivum L*.) in the Northern Great Plains. The point at which early seeding results in a decrease in grain yield has not been clearly identified. Changes in climatic conditions have increased frost-free periods and increased temperatures during grain filling, which can either be taken advantage of or avoided by seeding earlier. Field trials were conducted in western Canada from 2015 to 2018 to evaluate an ultra-early wheat planting system based on soil temperature triggers as opposed to calendar dates. Planting began when soil temperatures at 5 cm depth reached 0°C and continued at 2°C intervals until 10°C, regardless of calendar date. Conventional commercial spring wheat genetics and newly identified cold tolerant spring wheat lines were evaluated to determine if ultra-early wheat seeding systems required further development of specialized varieties to maintain system stability. Ultra-early seeding resulted in no detrimental effect on grain yield. Grain yield increased at sites south of 51° latitude N, and was unaffected by ultra-early seeding at sites north of 51° latitude N. Grain protein content, kernel weight, and bulk density were not affected by ultra-early seeding. Optimal seeding time was identified between 2 and 6°C soil temperatures. A greater reduction in grain yield was observed from delaying planting until soils reached 10°C than from seeding into 0°C soils; this was despite extreme environmental conditions after initial seeding, including air temperatures as low as −10.2°C, and as many as 37 nights with air temperatures below 0°C. Wheat emergence ranged from 55 to 70%, and heads m^−2^ decreased with delayed seeding while heads plant^−1^ did not change. Cold tolerant wheat lines did not increase stability of the ultra-early wheat seeding system relative to the conventional spring wheat check, and are therefore not required for growers to adopt ultra-early seeding. The results of this study indicate that growers in western Canada can successfully begin seeding wheat earlier, with few changes to their current management practices, and endure less risk than delaying seeding until soil temperatures reach 10°C or greater.

## Introduction

Canada is a globally important producer and exporter of high-quality wheat. In 2016 Canada ranked as the world's fifth largest producer of wheat (32.1 MT), and third largest exporter (19.7 MT) (FAOSTAT, 2019). Wheat production in the Northern Great Plains region of western Canada is limited by a short frost-free period which dictates the requirement for early maturing spring wheat varieties (Iqbal et al., 2007). Cutforth et al. (2004) calculated the average frost-free season in western Canada as 96 days in 1940, increasing to 114 days in 2000, a trend expected to continue. This increase in growing season length is one of many contributing factors accounting for increases in western Canadian spring wheat production from an average of 14.3 MT on 9.63 Mha from 1961 to 1970 to 19.8 MT on 6.63 Mha from 2008 to 2017 (Statistics Canada, 2019). Growth of the frost-free period has occurred as a result of both earlier final spring frosts, and later initial fall frosts (Cutforth et al., 2004), the former being correlated to calendar date and used as the current primary determinant of seeding date in western Canada. Lanning et al. (2010) reported that grain yield of the variety ‘Thatcher' had increased over 56 years of comparative data and attributed this to earlier planting and longer growing seasons. However, increased average growing season temperatures that have accompanied longer frost-free periods have the potential to reduce yield due to higher temperatures during grain fill and reduced in-season moisture availability (Asseng et al., 2004; Lanning et al., 2010; He et al., 2012). Kouadio et al. (2015) identified earlier seeding in western Canada as one method to reduce the risks associated with increased temperatures during the growing season as a result of climate change. In the evaluations of Kouadio et al. (2015) the lowest yield loss was observed from the earliest seeding dates. Many studies have indicated higher grain yield from seeding wheat earlier in western Canada (Larter et al., 1971; Mckenzie et al., 2008; McKenzie et al., 2011; Lanning et al., 2012) however, few have indicated the point at which seeding earlier has a detrimental effect on yield. Thilakarathna et al. (2017) evaluated frost-seeding, or seeding prior to spring ground thaw, in Ontario, Canada and determined a grain yield benefit of up to 24% over conventional seeding times for spring wheat in that environment, despite increased plant mortality.

The objective of this study was to evaluate an ultra-early spring wheat seeding system beginning at soil temperatures of 0°C. Ultra-early seeding treatments were combined with and without specialized cold-tolerant spring wheat genetics to determine if reductions in grain yield, grain quality, or growing system stability are associated with ultra-early seeding into cold soils in the Northern Great Plains.

## Materials and Methods

### Site Description, Experimental Design, and Seeding Date Determination

This study was conducted at five sites in western Canada over 4 years, 2015–2018, totaling 13 site years (**Table 1**). The treatment structure consisted of a factorial arrangement of 24 treatments based on four wheat lines, six planting dates, and four replicates blocked within replicate by planting date. The lines used were “AC Stettler” (DePauw et al., 2009), an industry standard Canada Western Red Spring (CWRS) wheat, and three cold tolerant experimental lines (LQ1282A, LQ1299A, LQ1315A) developed by intercrossing two previously identified cold tolerant lines derived from a cross between “Norstar” (Grant, 1980) Canada Western Red Winter (CWRW) wheat and “Bergen,” a Dark Northern Spring (DNS) wheat grown in North Dakota (**Table 2**). The seeding dates were based on soil temperature triggers of 0, 2, 4, 6, 8, and 10°C as measured with an Omega™ TPD42 soil temperature probe at 5 cm depth at 10:00 AM each day prior to seeding. If soil conditions made seeding impossible at the first soil temperature trigger (0°C), each seeding date was adjusted so that there was a 2°C soil temperature differential between each successive seeding date. The initial seeding date at each site in each year is shown in **Table 1**. In general, soil conditions at the sites south of 51°N allowed seeding to occur at targeted soil temperatures, while seeding at the sites north of 51°N began as soon as planting equipment could access trial sites and continued with 2°C soil temperature intervals between seeding dates. Access to trial sites at the higher latitude locations was often limited at 0°C soil temperatures due to excessive moisture and saturated soil after snow ablation.

**Table 1:** Average precipitation, post-seeding air temperature extremes and cumulative freezing events for each location x year.

Location	Latitude/longitude	Agroecological region	Soil zone	Average yearly precipitation† (mm)	Year	Actual precipitation (mm)	Earliest seeding date‡	Number of days with air temperature below 0°C after initial seeding date	Lowest air temperature recorded after seeding (°C)
Dawson Creek, BC	55°48'N 120°14'W	Parkland	Gray wooded	453	2015	325	April 16	12	−5.0
					2016	542	April 21	11	−6.1
Edmonton, AB	53°33'N 113°29'W	Parkland	Black	446	2015	299	April 9	12	−4.2
					2016	510	March 29	11	−3.6
					2017	416	May 5	0	2.3
Lethbridge, AB	49°41'N 112°50'W	Western Prairies	Dark brown	380	2015	251	March 6	37	−6.7
					2016	338	February 16	36	−10.2
					2017	249	March 20	17	−7.6
					2018	284	April 23	2	−1.2
Regina, SK	50°26'N 104°35'W	Western Prairies	Dark brown	397	2015	347	April 21	11	−5.0
Scott, SK	52°21'N 108°49'W	Western Prairies	Dark brown	366	2016	415	April 2	21	−9.8
					2017	300	March 31	27	−9.4
Swift Current, SK	50°18'N 107°46'W	Western Prairies	Brown	357	2015	304	April 10	23	−6.4

† 1981–2010 average yearly precipitation accumulation. ‡ Based on 0°C soil temperature trigger date.

**Table 2:** Classification of commercial check, cold tolerant, and parent lines.

Cultivar	Parental lines	Parental lines Canadian wheat classification	Experimental designation	Reference
AC Stettler^δ^	Prodigy, Superb	CWRS^δ^/CWRS	Commercial check	DePauw et al., 2009
LQ1282A^β^	Norstar, Bergen	CWRW^θ^/DNS^θ^	Cold tolerant^†^	Larsen, 2012
LQ1299A^β^	Norstar, Bergen	CWRW/DNS	Cold tolerant	Larsen, 2012
LQ1315A^β^	Norstar, Bergen	CWRW/DNS	Cold tolerant^†^	Larsen, 2012

† Cold tolerant lines were selected from 92 double haploid lines from a Norstar/Bergen cross initially completed at AAFC Lethbridge. Cold tolerant lines were selected based on demonstrated cold tolerance using LT_50_ tests as described in Larsen (2012). Further selection criteria included yield and quality parameters.

^δ^ Canada Western Red Spring

^θ^ Canada Western Red Winter

^θ^ Dark Northern Spring

^β^ Undetermined

### Cold Tolerant Wheat Lines

The cold-tolerant wheat lines used in this study were the result of work completed at Agriculture and Agri-Food Canada Lethbridge and the University of Guelph, where a proof of concept study successfully demonstrated the transfer of high levels of cold tolerance from Norstar winter wheat to spring wheat (Larsen, 2012). Briefly, spring growth habit, doubled haploid lines from a Bergen x Norstar cross were screened using an LT_50_ test to discover lines with exceptional cold tolerance. LT_50_ tests or lethal temperature tests, evaluate cold tolerance by identifying the temperature at which 50% mortality occurs among seedling plants (Fowler, 2008). Two of the best cold tolerant spring growth habit lines were intercrossed (A134$S10 x A134$S17) to develop lines with improved agronomics. Transfer of cold tolerance to spring wheat was successful, as several lines exhibited LT_50_ values superior to some commercial winter wheats commonly grown in eastern Canada. Thirty-nine semi-dwarf F_5:7_ derived cold tolerant lines were placed into a non-replicated preliminary yield trial established in Lethbridge in the fall of 2013 to identify superior lines. The same lines were increased and spring growth habit was confirmed at the Agriculture and Agri-Food Canada winter nursery in New Zealand over the winter of 2013/14. In the spring of 2014, both a yield trial and seed increase were established at Lethbridge to provide a robust data set of crop response variables (data not shown) which was used to select the three lines for this study (LQ1282A, LQ1299A, LQ1315A). In addition to cold tolerance, the selection criteria included high grain yield, grain protein content, and straw strength, and reduced plant height and maturity.

### Seeding Operations, Nutrient Management, and Pest Management

Seeding equipment varied but was similar to the drill designed and built by Agriculture and Agri-Food Canada Lethbridge, which was configured with ConservaPak™ knife openers (8) spaced 24 cm apart, a Valmar™ air delivery system, a Raven™ hydraulic seed calibration and product control system, and Morris™ seed cups. Fertilizer was banded to the side and below the seed row at seeding and was applied based on soil test recommendations and regional yield expectations. All seed was treated with a fungicide seed treatment to control seedling diseases [Raxil PRO—tebuconazole ({RS}-1-p-chlorophenyl-4,4-dimethyl-3-{1H-1,2,4-triazol-1-ylmethyl}pentan-3-ol] 3.0 g L^−1^ + prothioconazole [(RS)-2-[2-(1-chlorocyclopropyl)-3-(2-chlorophenyl)-2-hydroxypropyl]-2,4-dihydro-1,2,4-triazole-3-thione] 15.4 g L^−1^ + metalaxyl [metyl N-(methoxyacetyl)-N-2,6-xylyl-DL-alanite] 6.2 g L^−1^ Bayer Crop Science Canada Inc., Calgary, AB). All wheat lines were seeded at 400 viable seeds m^−2^.

Weed control was achieved with in-crop herbicide applications at the BBCH 12–22 stage of wheat, generally in late May. Due to variable staging between seeding dates, herbicide products with restrictive crop staging or residual properties were not used. All post-emergent herbicide applications were made using a motorized sprayer calibrated to deliver a carrier volume of 45 L ha^−1^ at 275 kPa pressure.

### Data Collection

Plant counts for each plot were performed from BBCH 20 to BBCH 49 to indicate total viable plants in two one-m long areas in the second and third rows and the second and third last rows of the plot. These areas were staked and used to count the number of heads later in the growing season. Heads plant^−1^ was calculated using the number of heads divided by the initial plant count for each staked area. Days to emergence were determined when 50% of plants in a plot had emerged. Crop anthesis was recorded in days from planting to when 50% of the heads in a plot began extruding anthers. Plant height was recorded from two randomly picked but representative areas of the plots and the height of five spikes, excluding awns, was measured.

The entire plot was harvested with a plot combine. The combine was equipped with a straight-cut header, pickup reel, and crop lifters. Grain yield per plot was weighed after drying the sample to 14% moisture content, and used to estimate total yield per ha (Mg Ha^−1^). A 2 kg subsample of grain was used to determine seed mass (from 250 kernels) and grain bulk density (kg hl^−1^). Whole grain protein concentration was determined from the same subsample using near infrared reflectance spectroscopy technology (Foss Decater GrainSpec, Foss Food Technology Inc, Eden Prairie, MN) (Irvine et al., 2013).

### Statistical Analyses

**Figure 1:**
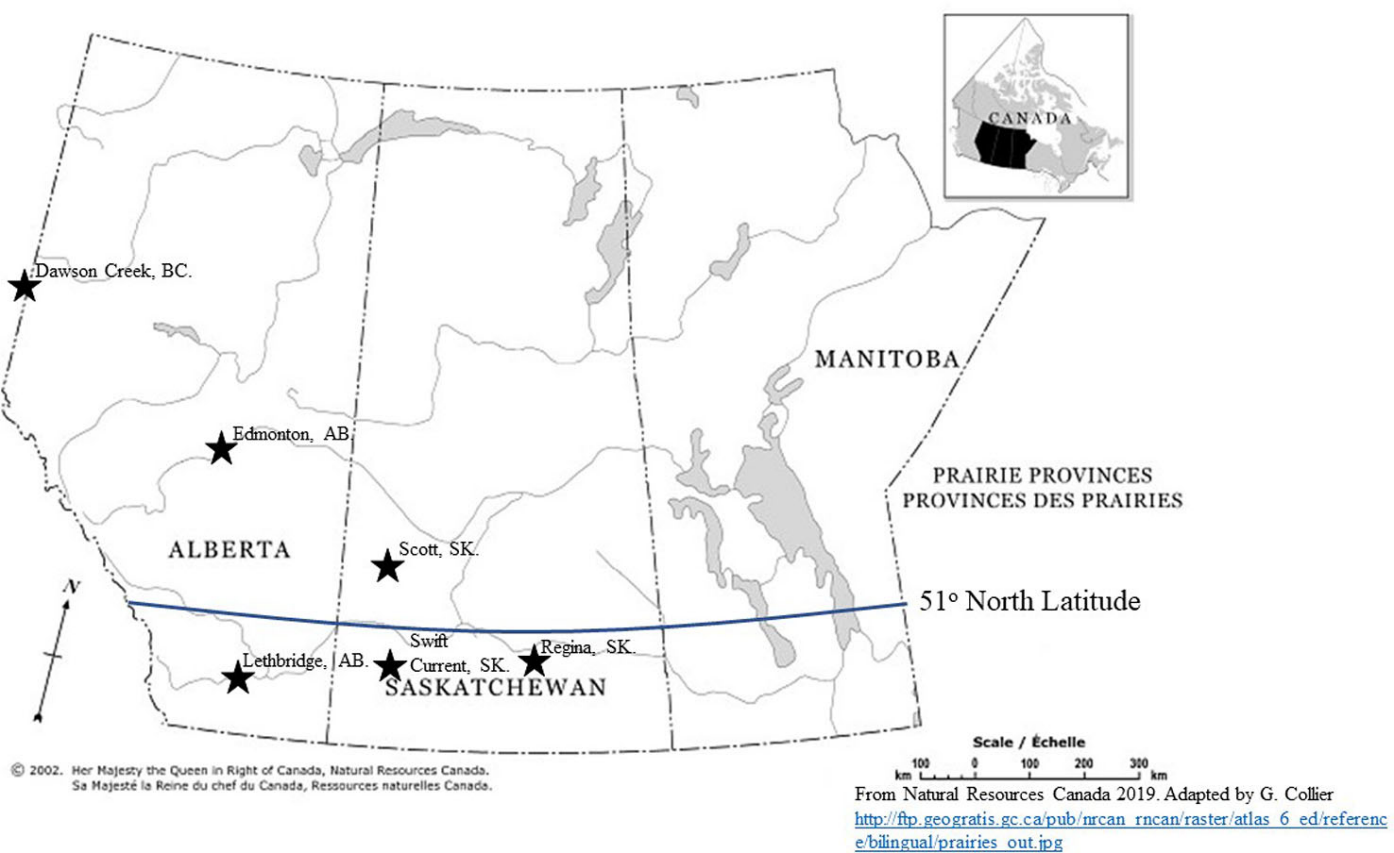
Geographical distribution of test locations for the assessment of ultra-early wheat planting in western Canada 2015 to 2018. (The Atlas of Canada—Natural Resources Canada. http://ftp.geogratis.gc.ca/pub/nrcan_rncan/raster/atlas_6_ed/reference/bilingual/prairies_out.jpg).

Data were analyzed in the MIXED procedure of SAS, and any outlier observations were removed before a combined analysis over years and environments (site-year) was performed using site-year, replication, soil temperature at seeding, and wheat variety as variables in the CLASS statement (Littell et al., 2006; SAS Institute, 2009). Error variances were heterogeneous among the environments, and corrected Akaike's information criterion (AICc) on model fit indicated that modeling residual variance heterogeneity improved fit. Variance heterogeneity was modeled for all analyses using the random statement in PROC MIXED with the group option set to environment. Environment and the interactions associated with environment were considered as random effects, whereas the treatment effects were considered fixed and significant if *P* ≤ 0.05 when the analysis was performed (Steel et al., 1997). Analyses were performed for environment groupings based on latitude. Sites north and south of 51° latitude were placed into two groups and analyzed separately (**Figure 1**).

The effect of planting date on yield was further evaluated with an analysis of covariance (ANCOVA) following the method developed by Yang and Juskiw (2011). The implementation of ANCOVA reduced the error mean square, accounted for missing data, and served to increase the precision of the resulting regression analysis. Planting date was considered a covariate and classification variable by generating a second column of data (s) identical to the planting date to be used as the covariate. Type 1 sums of squares was specified *via* the METHOD statement in PROC MIXED (Yang and Juskiw, 2011). Direct regression variables (covariates) s and s*s represent linear and quadratic responses to planting date and are part of the MODEL statement. Environment or group interactions with s and s*s are used to evaluate linear and quadratic responses that are heterogeneous relative to planting date. Initial ANCOVA analysis indicated a significant interaction between s*environment which supports the decision to analyze the environments in two groups based on latitude.

A biplot grouping methodology was used to explore system responses and variability of wheat yield as described by Francis and Kannenberg (1978). The mean and coefficient of variation (CV) across years and replications were estimated for each treatment combination. Means were plotted against CV, and used to categorize the biplot data into four quadrants/groups, which included high mean grain yield and low variability (group I), high mean grain yield and high variability (group II), low mean grain yield and high variability (group III), and low mean grain yield and low variability (group IV).

## Results and Discussion

### Growing Season Variability and Environmental Conditions

Initial seeding date varied within locations across years. Seeding began early in 2016—February 16 in Lethbridge, March 29 in Edmonton. The 2017 and 2018 seasons experienced delayed seeding due to late spring thaw. The first seeding date in Lethbridge in 2016 was 66 days earlier than the first seeding date in 2018. The first seeding date in Edmonton in 2016 was 37 days earlier than the first seeding date in 2017. Initial seeding dates by year and location are listed in **Table 1**. The wide range of environmental conditions that were experienced through the course of this study would be considered typical for the Northern Great Plains region as reported by Shen et al. (2005) who found no long term trends for the start of the growing season, defined as the first day of the year when five consecutive days have a mean temperature of 5°C, end of the growing season, the first day in the fall the mean temperature is below 5°C, and length of the growing season in the region. Shen et al. (2005) reported no change in the growing season despite reporting significant increases in frost free growing period, later first fall frosts, and earlier final spring frosts. The lack of an identifiable trend in growing season length, beginning, and end support the adoption of a soil-temperature-based seeding trigger system to standardize planting date from year to year and take advantage of the increased frost free period as opposed to the traditional use of calendar date as a reference point.

Precipitation varied over the duration of the study. Precipitation in 2015 was below average at all trial locations. In 2016 precipitation was above 30-year averages at all locations except Lethbridge, which was 89% of the 30-year average. All sites in 2017 and 2018 received below average rainfall (**Table 1**).

Eight of 13 site years experienced ambient air temperatures of −5°C or lower after initial seeding; some sites experienced temperatures as low as −9.8°C and −10.2°C. One site did not experience a nighttime low below 0°C after the initial seeding date. On average, sites had 16.5 nights where air temperatures reached below freezing, the most severe being Lethbridge in 2016 and 2017 where the air temperature dropped below 0°C for 37 and 36 nights respectively (**Table 1**).

After the initial planting date, nights with air temperatures below freezing averaged 21 at the sites south of 51°N and 12.5 at the sites north of 51°N. The soils at the sites south of 51°N tended to be free of snow cover and excess moisture and reached 0°C earlier than the sites north of 51°N. However, once sites north of 51°N reached 0°C they warmed faster than the sites south of 51°N, meaning planting dates were closer together at the sites north of 51°N (**Table 3**). This is due to the later disappearance of snow cover, increased available solar radiation when sites north of 51°N reached 0°C, and greater heat holding capacity of heavier texture clay loam soils relative to the sites south of 51°N (Zhao et al., 2002). The trial sites north of 51°N include soils classified as gray wooded Luvisols, orthic black chernozems, and orthic dark brown chernozems. The trial sites south of 51°N include soils of orthic dark brown chernozem and orthic brown chernozem classes.

**Table 3:** Least square means values and significance of crop development stage duration in ultra-early seeded wheat.

Planting date	Days to emergence	Days to anthesis	Days to maturity	Emergence to anthesis (days)	Anthesis to maturity (days)	Emergence to maturity (days)
**Sites South of 51°N latitude**^Ŧ^						
1 (Earliest)	23	79	140	56	60	117
2	22	82	138	60	56	116
3	21	76	136	56	60	115
4	18	76	135	58	59	117
5	13	69	131	55	62	117
6 (Latest)	13	65	125	53	60	113
**F-Test**	***	***	**	**	NS	NS
**SED**	1.6	2.6	3.9	1.8	NS	NS
**Linear**	***	***	***	*	NS	NS
**Quadratic**	NS	*	NS	*	NS	NS
**Sites North of 51°N latitude**^Ŧ^						
1	19	78	123	59	45	104
2	19	77	122	57	45	102
3	17	73	119	56	46	102
4	15	69	116	54	47	101
5	12	66	113	53	48	101
6	9	62	111	53	49	102
**F-Test**	***	***	***	***	*	*
**SED**	1.5	1.4	2.0	0.7	1.1	1.0
**Linear**	***	***	***	***	***	**
**Quadratic**	NS	NS	NS	*	NS	NS

(Ŧ) Data not reported for all environments. Sites south of 51° latitude include Lethbridge, AB. 2017, 2018. Sites north of 51° latitude include Dawson Creek, BC. 2016, Edmonton, AB. 2016, 2017, Scott, SK. 2016, 2017. (***) Significant at *P* < 0.001. (**) Significant at *P* < 0.01. (*) Significant at *P* < 0.05. (NS) Not significant. (SED) Standard error of the difference. (Ŧ) Planting date as determined by soil temperature trigger temperatures. Planting date (PD) 1 corresponds to a soil temperature of 0°C, or as soon after this trigger soil temperature as the site could be planted. Each successive PD corresponds to a 2°C increase in soil temperature from the previous PD.

Wheat emergence was slowed by the cool, slowly warming soils of sites south of 51°N. Wheat seeded at the earliest planting dates in the sites south of 51°N required 9.5 days longer to emerge than the earliest planting dates at the sites north of 51°N (**Table 3**).

### Seeding Date Effect

**Figure 2:**
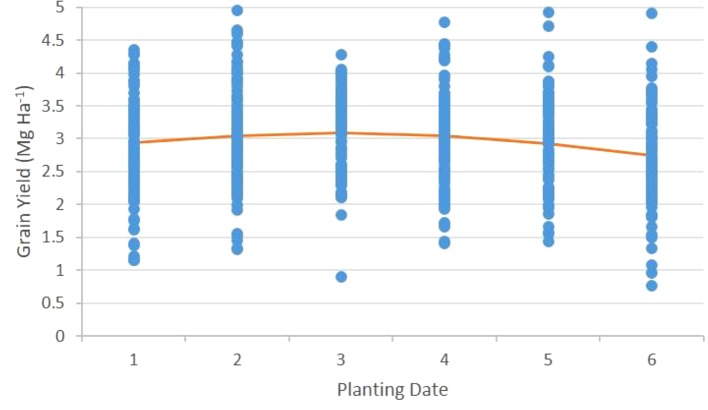
Wheat grain yield as a function of planting date (PD) in sites south of 51°N latitude. PD 1 corresponds to a soil temperature of 0°C, or as soon after this trigger soil temperature as the site could be planted. Each successive PD corresponds to a 2°C increase in soil temperature from the previous PD.) The line represents a quadratic regression for grain yield [yield = 2.7709 + (0.2125 × PD) − (0.03624 × PD^2^): *R^2^* = 0.61***] (***) Significant at *P* < 0.001.

Planting date did not alter yield at sites north of 51°N (*P = 0.158*) (**Table 4**). There was a yield response to planting date at sites south of 51°N (*P = 0.025*), and significant linear and quadratic effects of planting date on grain yield (*P = 0.044* and *P = 0.03* respectively) (**Tables 4** and **5**). The greatest grain yield occurred at the second and third planting dates, which correspond to soil temperature increase of 2 and 4°C after the earliest feasible planting date. Grain yield was lower at the earliest and latest seeding date (**Table 5**, **Figure 2**). The earliest seeding date produced less grain than the second and third seeding dates, however it did not produce less grain than the fourth or fifth seeding dates and yielded more grain than the latest seeding date. The optimum seeding time at sites south of 51°N in the Northern Great Plains of Canada is between soil temperatures of 2 and 6°C after the first possible seeding date (**Figure 2**). The regression equation determined in this study indicates a maximum grain yield is realized when seeding occurs prior to when soil temperatures reach 3.9°C. Planting as early as possible after soil temperature has reached 0°C, will result in the same grain yield as delaying seeding until soil temperatures warm by 7.7°C (**Figure 2**). Seeding after a soil temperature of 7.7°C above the first feasible seeding date will yield less grain than seeding as early as possible after ground thaw. Seeding dates prior to spring thaw as evaluated by Thilakaranthna et al. (2017) are often met with equipment and logistical restraints in western Canada. Seeding attempts prior to soil reaching 0°C in western Canada may be better served by fall seeding of winter wheat which has additional agronomic benefits as reviewed by Larsen et al. (2018).

**Table 4:** Probability values from the analysis of variance for each dataset for the fixed effects of planting date and wheat line. Environments, replicates within each environment, and interactions between random and fixed effects are considered to be random.

Effect	Yield (Mg ha^−1^)	Protein (%)	Test weight (kg hL^−1^)	Thousand Kernel weight (g)	Height (cm)	Heads m^−2^	Heads plant^−1^
**Sites South of 51°N latitude^θ^**							
Planting Date (PD)	**0.025**	0.072	0.67	0.75	0.051	**<0.001**	0.73
Wheat Line (WL)	**0.044**	**<0.001**	**<0.001**	**<0.001**	**0.047**	**0.023**	0.86
PD_linear_	**0.030**	**0.009**	0.54	0.26	**0.014**	**<0.001**	0.28
PD_quadratic_	**0.0047**	0.18	0.14	0.33	0.47	0.36	0.78
PD x WL	0.48	1.0	0.98	0.98	0.17	0.97	0.57
**Sites North of 51°N latitude^‡^**							
Planting date (PD)	0.16	0.21	0.60	0.091	**0.008**	0.28	0.14
Wheat line (WL)	**<0.001**	**<0.001**	**<0.001**	**<0.001**	**0.006**	**<0.001**	**<0.001**
PD_linear_	0.84	**0.015**	0.22	**0.005**	0.15	**0.039**	**0.028**
PD_quadratic_	0.22	0.33	0.52	0.86	**0.001**	0.31	0.54
PD x WL	0.95	0.91	0.94	1.0	0.81	0.96	0.88

θ 6 site years. Lethbridge, AB 2015, 2016, 2017, 2018. Regina, SK 2015, Swift Current, SK 2015

‡ 7 site years. Dawson Creek, BC 2015, 2016. Edmonton, AB 2015, 2016, 2017. Scott, SK 2016, 2017. Bolded values indicate a p - value of less than 0.05.

**Table 5:** Least square means values and significance of treatment interactions for sites south of 51°N latitude.

Planting date^Ŧ^	Yield (Mg ha^−1^)	Protein (%)	Test weight (kg hL^−1^)	Thousand Kernel weight (g)	Height (cm)	Heads m^−2^	Heads plant^−1^
1 (Earliest)	2.89	12.0	77.3	30.4	70	345	1.3
2	3.04	11.9	77.6	30.5	71	362	1.3
3	3.04	12.2	77.6	30.6	71	337	1.4
4	2.94	12.1	77.7	30.3	70	286	1.3
5	2.93	12.3	77.4	30.4	68	298	1.1
6 (Latest)	2.68	12.6	77.1	29.7	69	270	1.2
**F-Test**	*	NS	NS	NS	NS	***	NS
**SED**	0.12					56	
**Linear**	*	**	NS	NS	*	***	NS
**Quadratic**	**	NS	NS	NS	NS	NS	NS
							
**Wheat line**							
LQ1282A	3.00	11.5	77.3	28.2	70	318	1.3
LQ1299A	2.90	11.8	76.4	30.9	69	307	1.3
LQ1315A	2.91	11.8	77.2	30.4	70	309	1.3
AC Stettler	2.86	13.6	78.8	31.7	71	332	1.3
**F-Test**	*	***	***	***	*	*	NS
**SED**	0.06	0.14	0.2	0.3	1	11	
							
**Planting date x wheat line**	NS	NS	NS	NS	NS	NS	NS

(***) Significant at *P* < 0.001. (**) Significant at *P* < 0.01. (*) Significant at *P* < 0.05. (NS) Not significant. (SED) Standard error of the difference. (Ŧ) Planting date as determined by soil temperature trigger temperatures. Planting date (PD) 1 corresponds to a soil temperature of 0°C, or as soon after this trigger soil temperature as the site could be planted. Each successive PD corresponds to a 2°C increase in soil temperature from the previous PD.

Grain yield at sites south of 51°N is limited by lower precipitation and greater heat stress relative to sites located north of 51°N (**Table 1**, **Figure 2**). Delayed seeding at sites south of 51°N resulted in lower grain yield due to reduced water availability and daylight hours and increased temperature during critical grain fill periods (Farooq et al., 2011). Anthesis to maturity and emergence to maturity periods were not significantly different at any seeding dates at sites south of 51°N (**Table 3**). However, anthesis to maturity and emergence to maturity periods were offset as a result of seeding date. The length of days and available solar radiation captured in the anthesis to maturity and emergence to maturity periods of the earlier planting dates was greater than at the later planting dates.

Sites north of 51°N had no grain yield difference as a result of seeding date. Rapid soil temperature increases decreased time differential between each seeding date. Greater moisture availability and longer periods from anthesis to maturity compensated for potential grain yield loss associated with delayed seeding (**Table 3**).

Wheat is highly amenable to ultra-early seeding into cold soils; no negative yield effect relative to later plantings north or south of 51°N was discernable. Planting as early as possible had less negative effect on spring wheat yield than delaying seeding until soils had warmed 8 to 10°C (**Figure 2**).

Grain protein concentration, grain thousand kernel weight, and grain test weight were not affected by seeding date. Ultra-early seeding did not result in changes in grain quality despite increased grain yield in some environments. Previous studies have indicated increased grain yield is associated with decreased grain protein concentration (Iqbal et al., 2007a). Further evaluation is required to determine if ultra-early seeding can consistently result in greater grain yield without decreasing grain protein concentration.

Plant height was shorter at seeding dates two, three, and four at sites north of 51°N, while earlier and later seeding treatments were taller. The effect of seeding date on plant height had a significant positive quadratic association at sites north of 51°N (**Table 6**). Plant height at sites south of 51°N was not affected by seeding date.

**Table 6:** Least square means values and significance of treatment interactions for sites north of 51°N latitude.

Planting date ^Ŧ^	Yield (Mg ha^−1^)	Protein (%)	Test weight (kg hL^−1^)	Thousand Kernel weight (g)	Height (cm)	Heads m^−2^	Heads plant^−1^
1 (Earliest)	5.69	12.4	79.0	35.0	82	464	2.0
2	5.35	12.6	78.8	35.4	81	460	2.0
3	5.17	12.6	78.9	35.5	79	457	1.8
4	5.54	12.9	78.8	35.6	80	454	1.8
5	5.62	12.9	78.9	36.6	82	451	1.9
6 (Latest)	5.44	12.8	78.4	36.7	83	426	1.8
**F-Test**	NS	NS	NS	NS	**	NS	NS
**SED**					1		
**Linear**	NS	*	NS	**	NS	*	*
**Quadratic**	NS	NS	NS	NS	**	NS	NS
							
**Wheat line**							
LQ1282A	5.74	11.4	78.5	33.2	82	455	1.9
LQ1299A	5.48	12.2	78.1	36.4	80	417	1.7
LQ1315A	5.51	12.1	78.8	36.2	81	447	1.8
AC Stettler	5.14	15.2	79.8	37.4	81	491	2.0
**F-Test**	***	***	***	***	**	***	***
**SED**	0.10	0.2	0.2	0.3	1	7	0.1
							
**Planting date x wheat line**	NS	NS	NS	NS	NS	NS	NS

(***) Significant at *P* < 0.001. (**) Significant at *P* < 0.01. (*) Significant at *P* < 0.05. (NS) Not Significant. (SED) Standard error of the difference. (Ŧ) Planting date as determined by soil temperature trigger temperatures. Planting date (PD) 1 corresponds to a soil temperature of 0°C, or as soon after this trigger soil temperature as the site could be planted. Each successive PD corresponds to a 2°C increase in soil temperature from the previous PD.

The number of heads m^−2^ significantly decreased with delayed seeding at sites south of 51°N (**Table 4**). A significant negative linear effect for reduced number of heads m^−2^ and no significant difference in the number of heads plant^−1^, indicate that despite the extreme environmental conditions experienced when seeded ultra-early, the early planted wheat had better survivability than later seeded wheat, which did not initiate additional tillering to compensate for decreased plant stand (**Table 5**).

### Effect of Cold Tolerant Wheat Lines

Treatment effects were present for all reported variables in sites north and south of 51°N except for heads plant^−1^ in sites south of 51°N. Significant effects are the result of class differences between AC Stettler and the cold tolerant wheat lines LQ1282A, LQ1299A, and LQ1315A (**Table 2**). AC Stettler is a milling quality wheat of the CWRS class. The CWRS class wheats have high grain protein concentration, typically over 13.5%, which reduces grain yield potential (Iqbal et al., 2007a; Prairie Grain Development Committee, 2015). The cold tolerant lines used in this study are not registered varieties and have not been evaluated by the Prairie Grain Development Committee or the Canadian Grain Commission; however, the end-use characteristics of these lines indicate a likely classification of Canada Western Special Purpose (CWSP). CWSP wheats have reduced grain protein content and greater yield potential than CWRS wheats.

AC Stettler had higher grain protein content at all sites. At sites north of 51°N AC Stettler yielded less grain than the cold tolerant lines. At sites south of 51°N AC Stettler yielded less grain than LQ1282A, but yielded the same as LQ1299A, and LQ1315A (**Tables 5** and **6**). Grain yield of longer maturing cold tolerant wheat lines at sites south of 51°N may have been limited by reduced water availability and higher temperatures during grain fill. At sites south of 51°N AC Stettler had greater thousand kernel weight, grain test weight and heads m^−2^ values than the cold tolerant lines, additionally, there were no differences in heads plant^−1^ between AC Stettler and cold tolerant lines. Greater heads m^−2^ and no difference in heads plant^−1^ indicate the survival of AC Stettler was at least as good as the survival of the cold tolerant lines under ultra-early planting conditions.

### Ultra-Early Seeding System Stability

**Figure 3:**
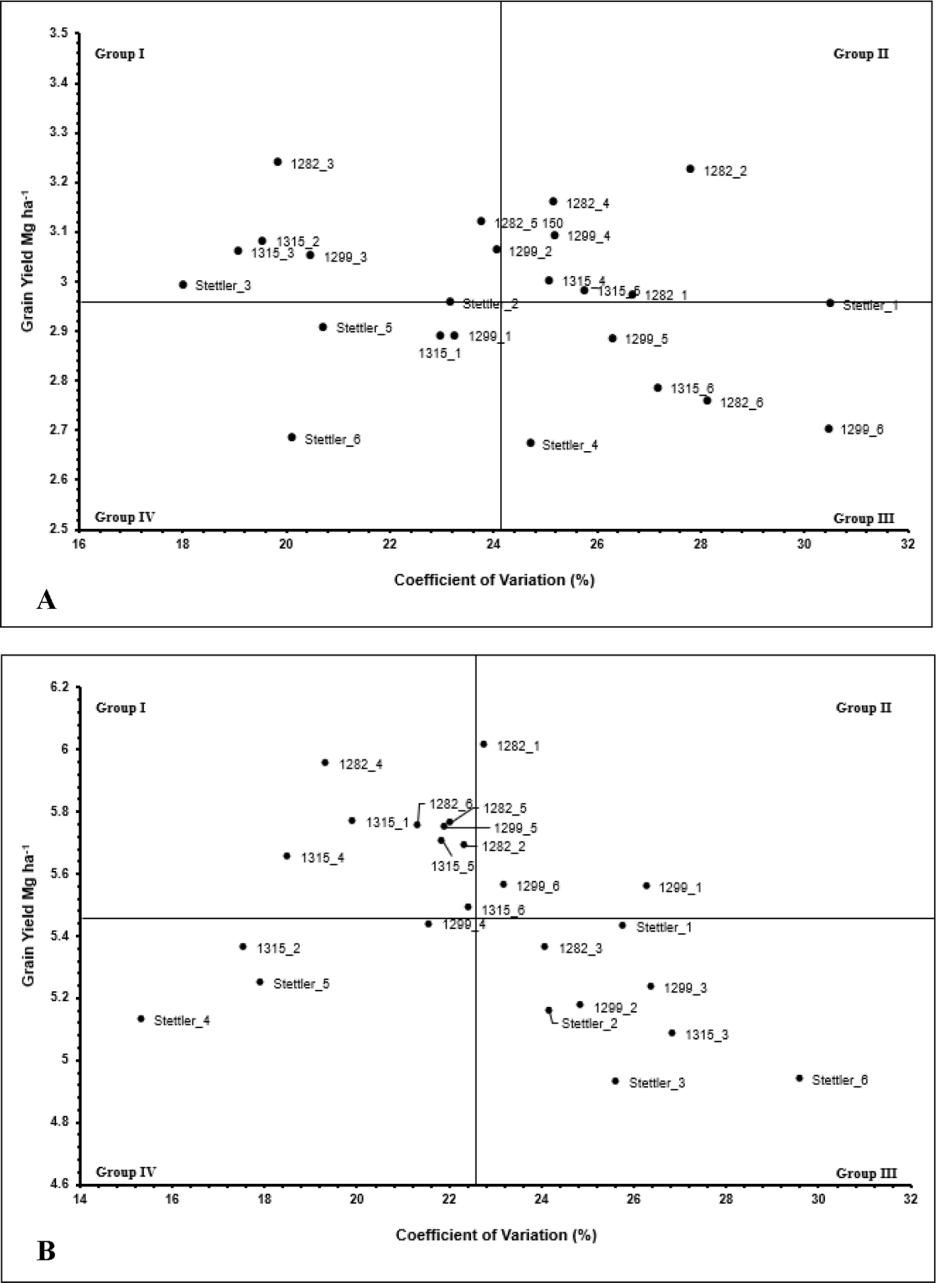
Biplots summarizing yield means *vs.* coefficient of variation (CV) for **(A)**: sites south of 51°N latitude, **(B)** sites north of 51°N latitude. Abbreviations are as follows: I) first number/name represents the wheat line (AC Stettler, LQ1282A, LQ1299A, and LQ1315A). II) Second number represents planting date (1–6). Grouping categories: group I: high mean, low variability; group II: high mean, high variability; group III: low mean, high variability; group IV: low mean, low variability.

A version of the Francis and Kannenberg (1978) biplot grouping method was used to help visualize the stability of ultra-early wheat seeding systems. The biplots for yield suggest advantages to an ultra-early seeding system in sites south of 51°N. Seeding dates two and three had the greatest yield and lowest variability (**Figure 3A**). Seeding date four maintained high grain yield, but variability increased at this seeding date. Seeding dates one, five, and six tended to result in higher variability and lower grain yield than seeding dates two, three, or four. AC Stettler consistently yielded less grain than the cold tolerant lines, but the stability of yield across seeding dates was similar to the cold tolerant varieties, as indicated by similar CV values.

Seeding date did not affect grain yield at sites north of 51°N. The biplot in **Figure 3B** shows mixed system stability responses to seeding date and grain yield. All seeding dates except seeding date three are represented by at least one data point in group I (high yield and low variability). Seeding ultra-early at sites north of 51°N did not reduce grain yield or system stability relative to delayed seeding.

An ultra-early wheat seeding system on the Northern Great Plains is feasible with few changes from current management systems. Grain yield was not negatively impacted by seeding very early and in some areas resulted in increased grain yield. At sites south of 51°N where seeding date where seeding date had a significant effect on yield, the earliest seeding date did not result in different grain yield from the fourth or fifth seeding date, and resulted in a higher grain yield than the final seeding date. We conclude that seeding spring wheat in the Northern Great Plains region of Canada can begin as soon as soil temperatures are above 0°C and seeding equipment can access fields. Ultra-early seeding did not result in decreased growing system stability or lower grain yield than delaying seeding until soil temperatures warmed 8 to 10°C. In sites south of 51°N growing system stability increased with ultra-early seeding.

The use of cold tolerant lines did not increase growing system stability relative to the conventional check variety AC Stettler. Based on studies by Fowler (2008), it was postulated that the ability of the cold tolerant spring wheat lines to acclimate to a relatively low LT_50_ would provide a useful genetic resource for enhanced cold temperature protection of commercial spring wheat varieties when seeded at ultra-early seeding dates, provided that there was adequate time for cold acclimation. These results, relative to AC Stettler, showed that for ultra-early spring wheat seeding, additional genetic cold temperature protection and increased rates of cold acclimation did not confer an advantage to the crop.

Currently in western Canada crop insurance systems maintain limits for the latest seeding dates a grower can plant a crop and receive compensation. The results of this study indicate that an incentive program to encourage early seeding may limit risk, increase grain yield potential, and increase growing system stability relative to current practices.

## Conclusions

Recommendations generated from this study are for growers to begin shifting to earlier spring wheat planting in western Canada—planting may begin immediately after the soil reaches 0°C, or as early as fields allow seeding operations to commence. A shift to seeding based on soil temperature triggers can normalize planting times within the growing season more effectively than seeding based on calendar date or on last expected spring frost. Special cold tolerant lines evaluated in this study did not benefit grain yield or stability of an ultra-early growing system. This study indicates that ultra-early seeding has no detrimental effect on yield on the Northern Great Plains and can potentially increase grain yield in lower latitude regions of western Canada. As indicated by Asseng et al. (2004); Lanning et al. (2010); He et al. (2012), and Kouadio et al. (2015), the risk of reduced grain yield in western Canada caused by increases in average growing season temperature and reduced precipitation can potentially be avoided by continually shifting wheat planting windows earlier. Future work will develop best management practices for an ultra-early wheat seeding system^©^ in western Canada and evaluate the benefits of optimized agronomic systems.

## Data Availability Statement

The datasets generated for this study are available on request to the corresponding author.

## Author Contributions

GC, Senior Author. Used project for partial fulfilment of PhD thesis requirements at the University of Alberta. Analyzed data and prepared manuscript. Participated in meetings and presented findings at conferences, field days and grower meetings. BB, Principal Investigator/Corresponding Author. Developed and conceptualized the hypotheses and field experiments. Identified and recruited Collaborators, PhD student, and led workshops to finalize proposal. Developed budget for all activities and co-supervises GC. RG, Co-Investigator. Developed cold-tolerant lines used for field experiments. Reviewed and edited manuscript. DS, Co-Investigator responsible for the field experimentation at the Edmonton sites. Co-Supervisor of GC. Reviewed and edited manuscript and mentored manuscript preparation and statistical analyses.

## Funding

This study was funded through the Agricultural Funding Consortium with funds provided by Alberta Innovates BioSolutions, the Alberta Wheat Commission, and the Western Grain Research Foundation Grant number 2014F172R.

## Conflict of Interest

The authors declare that the research was conducted in the absence of any commercial or financial relationships that could be construed as a potential conflict of interest.
